# 4-Chloro-*N*-(3,4-dichloro­phen­yl)-2-methyl­benzene­sulfonamide

**DOI:** 10.1107/S1600536811041717

**Published:** 2011-10-12

**Authors:** Vinola Z. Rodrigues, Sabine Foro, B. Thimme Gowda

**Affiliations:** a Department of Chemistry, Mangalore University, Mangalagangotri 574 199, Mangalore, India; b Institute of Materials Science, Darmstadt University of Technology, Petersenstrasse 23, D-64287 Darmstadt, Germany

## Abstract

In the title compound, C_13_H_10_Cl_3_NO_2_S, the N—C bond in the C—SO_2_—NH—C segment forms *trans* and *gauche* torsion angles with respect to the S=O bonds. Further, the N—H bond in the C—SO_2_—NH—C segment is *anti* to the *meta*-Cl atom in the anilino benzene ring and nearly *syn* with respect to the *ortho*-methyl group in the sulfonyl benzene ring. The C—SO_2_—NH—C torsion angle is −49.4 (2)°. The sulfonyl and aniline benzene rings are tilted relative to each other by 54.6 (1)°. In the crystal, mol­ecules are linked into chains along the *c*-axis direction by inter­molecular N—H⋯O hydrogen bonds.

## Related literature

For the preparation of the title compound, see: Savitha & Gowda (2006 ▶). For hydrogen-bonding modes of sulfonamides, see: Adsmond & Grant (2001 ▶). For studies on the effects of substituents on the structures and other aspects of *N*-(ar­yl)-amides, see: Gowda *et al.* (2003 ▶), on *N*-(ar­yl)-methane­sulfonamides, see: Gowda *et al.* (2007 ▶), on *N*-(ar­yl)-aryl­sulfonamides, see: Gelbrich *et al.* (2007 ▶); Perlovich *et al.* (2006 ▶); Rodrigues *et al.* (2011 ▶); Shetty & Gowda (2005 ▶) and on *N*-(chloro)-aryl­sulfonamides, see: Gowda & Kumar (2003 ▶).
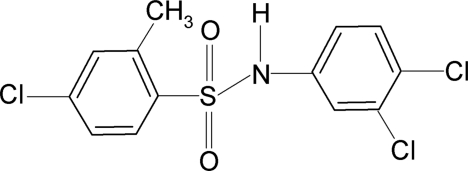


## Experimental

### 

#### Crystal data

C_13_H_10_Cl_3_NO_2_S
*M* *_r_* = 350.63
Monoclinic, 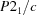

*a* = 14.563 (2) Å
*b* = 10.033 (2) Å
*c* = 10.162 (2) Å
β = 92.60 (2)°
*V* = 1483.2 (5) Å^3^
*Z* = 4
Mo *K*α radiation
μ = 0.76 mm^−1^
*T* = 293 K
0.44 × 0.44 × 0.38 mm

#### Data collection

Oxford Diffraction Xcalibur diffractometer with a Sapphire CCD detector
Absorption correction: multi-scan (*CrysAlis RED*; Oxford Diffraction, 2009 ▶) *T*_min_ = 0.732, *T*_max_ = 0.762
5266 measured reflections
3005 independent reflections
2373 reflections with *I* > 2σ(*I*)
*R*_int_ = 0.021

#### Refinement

*R*[*F* ^2^ > 2σ(*F* ^2^)] = 0.045
*wR*(*F* ^2^) = 0.126
*S* = 1.05
3005 reflections
185 parameters
1 restraint
H atoms treated by a mixture of independent and constrained refinement
Δρ_max_ = 0.41 e Å^−3^
Δρ_min_ = −0.42 e Å^−3^

### 

Data collection: *CrysAlis CCD* (Oxford Diffraction, 2009 ▶); cell refinement: *CrysAlis CCD*; data reduction: *CrysAlis RED* (Oxford Diffraction, 2009 ▶); program(s) used to solve structure: *SHELXS97* (Sheldrick, 2008 ▶); program(s) used to refine structure: *SHELXL97* (Sheldrick, 2008 ▶); molecular graphics: *PLATON* (Spek, 2009 ▶); software used to prepare material for publication: *SHELXL97*.

## Supplementary Material

Crystal structure: contains datablock(s) I, global. DOI: 10.1107/S1600536811041717/tk2797sup1.cif

Structure factors: contains datablock(s) I. DOI: 10.1107/S1600536811041717/tk2797Isup2.hkl

Supplementary material file. DOI: 10.1107/S1600536811041717/tk2797Isup3.cml

Additional supplementary materials:  crystallographic information; 3D view; checkCIF report

## Figures and Tables

**Table 1:** Hydrogen-bond geometry (Å, °)

*D*—H⋯*A*	*D*—H	H⋯*A*	*D*⋯*A*	*D*—H⋯*A*
N1—H1*N*⋯O1^i^	0.85 (2)	2.11 (2)	2.868 (3)	149 (3)

Symmetry code: (i) 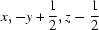.

**Fig. 1.**
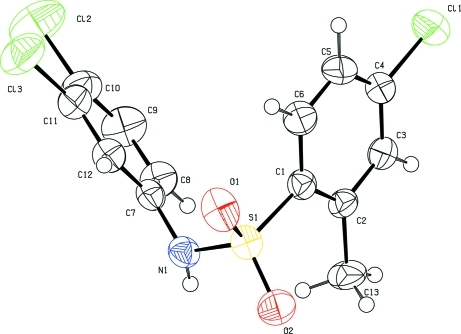
Molecular structure of the title compound, showing the atom labelling scheme and displacement ellipsoids drawn at the 50% probability level.

**Fig. 2.**
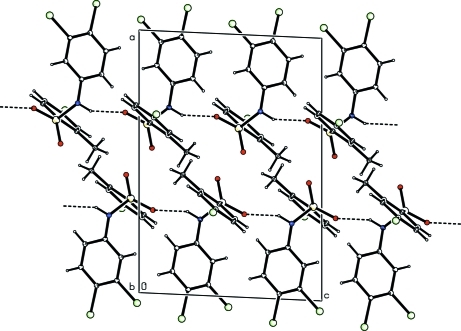
Molecular packing of (I) with hydrogen bonding shown as dashed lines.

## supplementary crystallographic information

### Comment

The sulfonamide moiety is the constituent of many biologically significant compounds. The hydrogen bonding preferences of sulfonamides have been investigated (Adsmond & Grant, 2001). As part of our studies on the substituent effects on the structures and other aspects of *N*-(aryl)-amides (Gowda *et al.*, 2003), *N*-(aryl)-methanesulfonamides (Gowda *et al.*, 2007), *N*-(aryl)-arylsulfonamides (Rodrigues *et al.*, 2011; Shetty & Gowda, 2005) and *N*-(chloro)-arylsulfonamides (Gowda & Kumar, 2003), in the present work, the crystal structure of 4-chloro-2-methyl-*N*-(3,4-dichlorophenyl)benzenesulfonamide (I) has been determined (Fig. 1).

In (I), the conformation of the N—C bond in the C—SO_2_—NH—C segment has *trans* and *gauche* torsions with respect to the S═O bonds. Further, the N—H bond in the C—SO_2_—NH—C segment is *anti* with respect to the *meta*-Cl atom in the anilino benzene ring and nearly *syn* with respect to the *ortho*-methyl group in the sulfonyl benzene ring. The molecule is bent at the S atom with the C—SO_2_—NH—C torsion angle of -49.42 (23)°, compared to the value of -49.72 (18)° in 4-Chloro-2-methyl-*N*-(3,4-dimethylphenyl)- benzenesulfonamide (II) (Rodrigues *et al.*, 2011).

The sulfonyl and the aniline benzene rings are tilted relative to each other by 54.6 (1)°, compared to the values of 71.6 (1)° in (II).

The other bond parameters in (I) are similar to those observed in (II) and other aryl sulfonamides (Perlovich *et al.*, 2006; Gelbrich *et al.*, 2007).

In the crystal, the intermolecular N–H···O hydrogen bonds (Table 1) link the molecules into infinite chains. Part of the crystal structure is shown in Fig. 2.

### Experimental

The solution of *m*-chlorotoluene (10 ml) in chloroform (40 ml) was treated drop wise with chlorosulfonic acid (25 ml) at 273 K. After the initial evolution of hydrogen chloride subsided, the reaction mixture was brought to room temperature and poured into crushed ice in a beaker. The chloroform layer was separated, washed with cold water and allowed to evaporate slowly. The residual 2-methyl-4-chlorobenzenesulfonylchloride was treated with a stoichiometric amount of 3,4-dichloroaniline and boiled for ten minutes. The reaction mixture was then cooled to room temperature and added to ice-cold water (100 ml). The resultant solid, 4-chloro-2-methyl-*N*- (3,4-dichlorophenyl)benzenesulfonamide, was filtered under suction and washed thoroughly with cold water. It was then recrystallized to constant melting point from dilute ethanol (Savitha & Gowda, 2006). Colourless prisms were grown from its ethanol solution by slow evaporation at room temperature.

### Refinement

The NH H atom was located in a difference map and later restrained to N—H = 0.86±0.02 Å. The other H atoms were positioned with idealized geometries using a riding model with the aromatic-C—H = 0.93 Å and methyl-C—H = 0.96 Å. The *U*_iso_(H) values were set at 1.2*U*_eq_(C-aromatic, N) and 1.5*U*_eq_(*C*-methyl).

### Crystal data

**Table:**

C_13_H_10_Cl_3_NO_2_S	*F*(000) = 712
*M**_r_* = 350.63	*D*_x_ = 1.570 Mg m^−^^3^
Monoclinic, *P*2_1_/*c*	Mo *K*α radiation, λ = 0.71073 Å
Hall symbol: -P 2ybc	Cell parameters from 926 reflections
*a* = 14.563 (2) Å	θ = 2.9–27.8°
*b* = 10.033 (2) Å	µ = 0.76 mm^−^^1^
*c* = 10.162 (2) Å	*T* = 293 K
β = 92.60 (2)°	Prism, colourless
*V* = 1483.2 (5) Å^3^	0.44 × 0.44 × 0.38 mm
*Z* = 4	

### Data collection

**Table:**

Oxford Diffraction Xcalibur diffractometer with a Sapphire CCD detector	3005 independent reflections
Radiation source: fine-focus sealed tube	2373 reflections with *I* > 2σ(*I*)
graphite	*R*_int_ = 0.021
Rotation method data acquisition using ω scans	θ_max_ = 26.4°, θ_min_ = 2.9°
Absorption correction: multi-scan (*CrysAlis RED*; Oxford Diffraction, 2009)	*h* = −18→17
*T*_min_ = 0.732, *T*_max_ = 0.762	*k* = −7→12
5266 measured reflections	*l* = −11→12

### Refinement

**Table:**

Refinement on *F*^2^	Primary atom site location: structure-invariant direct methods
Least-squares matrix: full	Secondary atom site location: difference Fourier map
*R*[*F*^2^ > 2σ(*F*^2^)] = 0.045	Hydrogen site location: inferred from neighbouring sites
*wR*(*F*^2^) = 0.126	H atoms treated by a mixture of independent and constrained refinement
*S* = 1.05	*w* = 1/[σ^2^(*F*_o_^2^) + (0.0556*P*)^2^ + 1.2273*P*] where *P* = (*F*_o_^2^ + 2*F*_c_^2^)/3
3005 reflections	(Δ/σ)_max_ < 0.001
185 parameters	Δρ_max_ = 0.41 e Å^−^^3^
1 restraint	Δρ_min_ = −0.42 e Å^−^^3^

### Special details

**Table:**

Geometry. All e.s.d.'s (except the e.s.d. in the dihedral angle between two l.s. planes) are estimated using the full covariance matrix. The cell e.s.d.'s are taken into account individually in the estimation of e.s.d.'s in distances, angles and torsion angles; correlations between e.s.d.'s in cell parameters are only used when they are defined by crystal symmetry. An approximate (isotropic) treatment of cell e.s.d.'s is used for estimating e.s.d.'s involving l.s. planes.
Refinement. Refinement of *F*^2^ against ALL reflections. The weighted *R*-factor *wR* and goodness of fit *S* are based on *F*^2^, conventional *R*-factors *R* are based on *F*, with *F* set to zero for negative *F*^2^. The threshold expression of *F*^2^ > σ(*F*^2^) is used only for calculating *R*-factors(gt) *etc*. and is not relevant to the choice of reflections for refinement. *R*-factors based on *F*^2^ are statistically about twice as large as those based on *F*, and *R*- factors based on ALL data will be even larger.

### Fractional atomic coordinates and isotropic or equivalent isotropic displacement parameters (Å^2^)

**Table:**

	*x*	*y*	*z*	*U*_iso_*/*U*_eq_	
C1	0.35205 (17)	0.5208 (3)	0.4395 (2)	0.0366 (5)	
C2	0.39656 (16)	0.5884 (3)	0.3403 (2)	0.0376 (5)	
C3	0.38485 (18)	0.7259 (3)	0.3330 (3)	0.0426 (6)	
H3	0.4140	0.7740	0.2688	0.051*	
C4	0.33077 (18)	0.7916 (3)	0.4196 (3)	0.0445 (6)	
C5	0.2867 (2)	0.7245 (3)	0.5166 (3)	0.0517 (7)	
H5	0.2505	0.7703	0.5746	0.062*	
C6	0.2973 (2)	0.5885 (3)	0.5262 (3)	0.0487 (7)	
H6	0.2677	0.5416	0.5909	0.058*	
C7	0.19793 (17)	0.3372 (3)	0.3148 (2)	0.0384 (6)	
C8	0.1756 (2)	0.4145 (3)	0.2057 (3)	0.0519 (7)	
H8	0.2208	0.4376	0.1480	0.062*	
C9	0.0865 (2)	0.4577 (4)	0.1820 (3)	0.0656 (9)	
H9	0.0715	0.5090	0.1079	0.079*	
C10	0.0196 (2)	0.4247 (4)	0.2682 (3)	0.0597 (8)	
C11	0.0421 (2)	0.3503 (3)	0.3786 (3)	0.0517 (7)	
C12	0.13136 (19)	0.3057 (3)	0.4024 (3)	0.0454 (6)	
H12	0.1464	0.2549	0.4768	0.054*	
C13	0.4565 (2)	0.5208 (3)	0.2399 (3)	0.0542 (8)	
H13A	0.4225	0.4502	0.1966	0.065*	
H13B	0.5106	0.4847	0.2843	0.065*	
H13C	0.4742	0.5850	0.1757	0.065*	
Cl1	0.31845 (6)	0.96380 (8)	0.40549 (10)	0.0668 (3)	
Cl2	−0.09220 (7)	0.47671 (15)	0.23365 (13)	0.1025 (4)	
Cl3	−0.04016 (6)	0.30826 (11)	0.48889 (11)	0.0810 (3)	
N1	0.28934 (15)	0.2871 (2)	0.3357 (2)	0.0413 (5)	
H1N	0.3167 (19)	0.274 (3)	0.265 (2)	0.050*	
O1	0.31664 (17)	0.3122 (2)	0.57571 (19)	0.0578 (6)	
O2	0.44650 (14)	0.2959 (2)	0.4296 (2)	0.0573 (5)	
S1	0.35718 (5)	0.34532 (7)	0.45424 (6)	0.04128 (19)	

### Atomic displacement parameters (Å^2^)

**Table:**

	*U*^11^	*U*^22^	*U*^33^	*U*^12^	*U*^13^	*U*^23^
C1	0.0362 (12)	0.0393 (13)	0.0344 (12)	−0.0026 (10)	0.0007 (10)	−0.0030 (10)
C2	0.0325 (11)	0.0432 (14)	0.0376 (12)	−0.0018 (11)	0.0069 (10)	−0.0038 (11)
C3	0.0389 (13)	0.0426 (14)	0.0469 (14)	−0.0051 (11)	0.0084 (11)	0.0019 (12)
C4	0.0405 (13)	0.0389 (14)	0.0540 (16)	−0.0015 (11)	0.0014 (12)	−0.0059 (12)
C5	0.0524 (16)	0.0505 (17)	0.0537 (16)	0.0012 (13)	0.0183 (13)	−0.0147 (14)
C6	0.0545 (16)	0.0531 (17)	0.0398 (14)	−0.0078 (14)	0.0172 (12)	−0.0049 (13)
C7	0.0432 (13)	0.0350 (13)	0.0369 (13)	−0.0034 (11)	0.0004 (10)	−0.0055 (10)
C8	0.0549 (16)	0.0552 (18)	0.0458 (15)	0.0018 (14)	0.0053 (13)	0.0072 (14)
C9	0.069 (2)	0.074 (2)	0.0538 (18)	0.0123 (18)	−0.0016 (16)	0.0139 (17)
C10	0.0492 (16)	0.066 (2)	0.0632 (19)	0.0130 (15)	−0.0027 (15)	−0.0027 (16)
C11	0.0478 (15)	0.0518 (17)	0.0559 (17)	−0.0030 (13)	0.0079 (13)	−0.0032 (14)
C12	0.0496 (15)	0.0443 (15)	0.0423 (14)	−0.0013 (12)	0.0025 (12)	0.0010 (12)
C13	0.0536 (16)	0.0486 (16)	0.0631 (18)	0.0065 (14)	0.0322 (15)	0.0066 (14)
Cl1	0.0696 (5)	0.0436 (4)	0.0880 (6)	0.0018 (4)	0.0133 (4)	−0.0057 (4)
Cl2	0.0603 (5)	0.1361 (11)	0.1105 (9)	0.0337 (6)	−0.0036 (6)	0.0203 (8)
Cl3	0.0575 (5)	0.0977 (7)	0.0900 (7)	0.0007 (5)	0.0267 (5)	0.0084 (6)
N1	0.0458 (12)	0.0412 (12)	0.0369 (11)	−0.0017 (10)	0.0039 (9)	−0.0053 (10)
O1	0.0820 (15)	0.0555 (12)	0.0357 (10)	−0.0111 (11)	0.0012 (10)	0.0099 (9)
O2	0.0465 (11)	0.0550 (12)	0.0693 (14)	0.0099 (10)	−0.0077 (10)	0.0041 (11)
S1	0.0465 (4)	0.0410 (4)	0.0360 (3)	−0.0022 (3)	−0.0017 (3)	0.0034 (3)

### Geometric parameters (Å, °)

**Table:**

C1—C6	1.392 (4)	C8—H8	0.9300
C1—C2	1.399 (4)	C9—C10	1.380 (5)
C1—S1	1.768 (3)	C9—H9	0.9300
C2—C3	1.392 (4)	C10—C11	1.375 (4)
C2—C13	1.531 (4)	C10—Cl2	1.730 (3)
C3—C4	1.375 (4)	C11—C12	1.386 (4)
C3—H3	0.9300	C11—Cl3	1.729 (3)
C4—C5	1.376 (4)	C12—H12	0.9300
C4—Cl1	1.743 (3)	C13—H13A	0.9600
C5—C6	1.377 (4)	C13—H13B	0.9600
C5—H5	0.9300	C13—H13C	0.9600
C6—H6	0.9300	N1—S1	1.631 (2)
C7—C8	1.380 (4)	N1—H1N	0.847 (17)
C7—C12	1.382 (4)	O1—S1	1.431 (2)
C7—N1	1.430 (3)	O2—S1	1.425 (2)
C8—C9	1.378 (4)		
			
C6—C1—C2	121.2 (3)	C10—C9—H9	120.0
C6—C1—S1	117.1 (2)	C11—C10—C9	119.9 (3)
C2—C1—S1	121.61 (19)	C11—C10—Cl2	121.1 (3)
C3—C2—C1	117.3 (2)	C9—C10—Cl2	119.0 (3)
C3—C2—C13	118.4 (2)	C10—C11—C12	120.3 (3)
C1—C2—C13	124.3 (2)	C10—C11—Cl3	121.0 (2)
C4—C3—C2	120.9 (3)	C12—C11—Cl3	118.7 (2)
C4—C3—H3	119.5	C7—C12—C11	119.6 (3)
C2—C3—H3	119.5	C7—C12—H12	120.2
C3—C4—C5	121.5 (3)	C11—C12—H12	120.2
C3—C4—Cl1	118.9 (2)	C2—C13—H13A	109.5
C5—C4—Cl1	119.6 (2)	C2—C13—H13B	109.5
C4—C5—C6	118.8 (3)	H13A—C13—H13B	109.5
C4—C5—H5	120.6	C2—C13—H13C	109.5
C6—C5—H5	120.6	H13A—C13—H13C	109.5
C5—C6—C1	120.3 (3)	H13B—C13—H13C	109.5
C5—C6—H6	119.9	C7—N1—S1	120.79 (18)
C1—C6—H6	119.9	C7—N1—H1N	114 (2)
C8—C7—C12	120.0 (3)	S1—N1—H1N	113 (2)
C8—C7—N1	120.0 (2)	O2—S1—O1	119.09 (14)
C12—C7—N1	120.0 (2)	O2—S1—N1	105.82 (13)
C9—C8—C7	120.2 (3)	O1—S1—N1	107.11 (13)
C9—C8—H8	119.9	O2—S1—C1	111.54 (13)
C7—C8—H8	119.9	O1—S1—C1	106.65 (13)
C8—C9—C10	120.0 (3)	N1—S1—C1	105.81 (12)
C8—C9—H9	120.0		
			
C6—C1—C2—C3	0.9 (4)	Cl2—C10—C11—C12	177.8 (2)
S1—C1—C2—C3	177.02 (19)	C9—C10—C11—Cl3	179.6 (3)
C6—C1—C2—C13	−178.9 (3)	Cl2—C10—C11—Cl3	−1.2 (4)
S1—C1—C2—C13	−2.8 (4)	C8—C7—C12—C11	1.1 (4)
C1—C2—C3—C4	−0.7 (4)	N1—C7—C12—C11	−177.4 (2)
C13—C2—C3—C4	179.1 (3)	C10—C11—C12—C7	0.4 (5)
C2—C3—C4—C5	0.3 (4)	Cl3—C11—C12—C7	179.4 (2)
C2—C3—C4—Cl1	−179.8 (2)	C8—C7—N1—S1	109.5 (3)
C3—C4—C5—C6	−0.2 (4)	C12—C7—N1—S1	−72.0 (3)
Cl1—C4—C5—C6	180.0 (2)	C7—N1—S1—O2	−167.9 (2)
C4—C5—C6—C1	0.4 (4)	C7—N1—S1—O1	64.1 (2)
C2—C1—C6—C5	−0.8 (4)	C7—N1—S1—C1	−49.4 (2)
S1—C1—C6—C5	−177.1 (2)	C6—C1—S1—O2	−143.8 (2)
C12—C7—C8—C9	−1.7 (5)	C2—C1—S1—O2	40.0 (2)
N1—C7—C8—C9	176.9 (3)	C6—C1—S1—O1	−12.2 (2)
C7—C8—C9—C10	0.7 (5)	C2—C1—S1—O1	171.5 (2)
C8—C9—C10—C11	0.8 (5)	C6—C1—S1—N1	101.6 (2)
C8—C9—C10—Cl2	−178.4 (3)	C2—C1—S1—N1	−74.6 (2)
C9—C10—C11—C12	−1.4 (5)		

### Hydrogen-bond geometry (Å, °)

**Table:**

*D*—H···*A*	*D*—H	H···*A*	*D*···*A*	*D*—H···*A*
N1—H1N···O1^i^	0.85 (2)	2.11 (2)	2.868 (3)	149 (3)

Symmetry codes: (i) *x*, −*y*+1/2, *z*−1/2.
